# Fall armyworm in Botswana: impacts, farmer management practices and implications for sustainable pest management

**DOI:** 10.1002/ps.6717

**Published:** 2021-12-02

**Authors:** Fernadis Makale, Idah Mugambi, Monica K Kansiime, Irie Yuka, Mathew Abang, Bonolo S Lechina, Mosimanegape Rampeba, Ivan Rwomushana

**Affiliations:** ^1^ CAB International, Africa Regional Centre Nairobi Kenya; ^2^ FAO Botswana Gaborone Botswana; ^3^ FAO Sub regional Office for Southern Africa Harare Zimbabwe; ^4^ Ministry of Agricultural Development and Food Security (MoA) Gaborone Botswana

**Keywords:** fall armyworm, impacts, farmer management practices, food self‐sufficiency, Botswana

## Abstract

**BACKGROUND:**

Since first reported in Botswana, fall armyworm (FAW) continues to be a threat to crop production. This study aimed to estimate impacts of FAW on yield and farmers' livelihoods in Botswana, and to obtain data that could be extrapolated to national level. Further, farmer knowledge of the pest, management practices and pesticide use for FAW management were assessed.

**RESULTS:**

In fact, 76% of the 220 respondents had seen FAW in their farms in the 2018/2019 cropping season, affecting almost the entire and about half of cultivated area for maize and sorghum, respectively. Thus, 51% of the respondents implemented FAW control measures, with chemical pesticides (27%) being the most common management against FAW. Only 33% of respondents in 2018/2019 were food self‐sufficient, as opposed to 80% in an ordinary year, with farmers who reported not to have been affected by FAW more likely to be insufficient with food (88%) compared to 60% of the farmers who reported FAW attack. Drought was ranked the major stress experienced by the famers (35%), and also showed significant yield reducing effects on maize yield with pest and diseases reported second most important. Pesticides (20%) and training on pest management (18%) were the top ranked needs by farmers interviewed.

**CONCLUSIONS:**

This study confirms the impact and threat of FAW to crop production in Botswana. Chemicals remain the go‐to control option by a majority of the farmers. Other low‐risk technologies exist and are proposed for adoption in the management of FAW. Of note is the acknowledgement that a single control strategy will not be effective against FAW and as such integrated pest management (IPM) on an area‐wide scale is needed to achieve best results. Mass awareness, training and demonstration will be required to achieve this.

## Graphical abstract

Pesticides were the commonest control measures against fall armyworm (FAW). Drought was ranked the top stress with pesticides and training in pest management being the important needs prioritized by farmers.

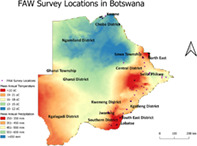

© 2021 The Authors. *Pest Management Science* published by John Wiley & Sons Ltd on behalf of Society of Chemical Industry.

## 1 INTRODUCTION

Botswana is a landlocked country in southern Africa. It is topographically flat, tending toward gently rolling tableland with 70% of its territory being Kalahari Desert. Formerly one of the poorest countries in the world, with a gross domestic product (GDP) *per capita* of about US$70 in the late 1960s, Botswana has since transformed itself into an upper middle‐income country with one of the fastest growing economies. By one estimate, it has the fourth highest gross national income at purchasing power parity in Africa.^1^ The economy is dominated by mining, livestock keeping and tourism. The mineral industry provides about 40% of all government revenues^2^, ^3^ with diamond, gold, uranium, copper, and even oil being among the minerals present in Botswana.

Botswana is faced by two major environmental problems, drought and desertification, which are heavily linked. Three‐quarters of the country's human and animal populations depend on groundwater due to drought.^4^ Surface water is scarce in Botswana and only less than 5% of the agriculture in the country is rainfed. In the remaining 95% of the country, raising livestock is the primary source of rural income.^5^ Approximately 71% of the country's land is used for communal grazing,^6^ and this has been a major cause of the desertification and the accelerating soil erosion of the country. However, with the profitability of raising livestock, land exploitation continues.

Agriculture plays an important role in rural development by providing food, income and employment for the majority of the rural dwellers. In Botswana, agriculture is practiced in two distinct sectors, namely the commercial and traditional sectors and accounts for about 3% of Botswana's GDP.^7^, ^8^ However, Botswana's agricultural potential is, unarguably, limited. The Kalahari Desert occupies a large area of the country, and recent regional droughts have not helped the areas where rain‐fed agriculture is the norm. Only about 0.7% of total land area is arable,^9^ most of which is in the eastern region. Here, sorghum, millet and maize are the main subsistence crops, with groundnuts, beans and sunflower also grown. With the high demand for these foods/crops compared to the production/supply, occasional importation by the government from neighbouring Zimbabwe and South Africa happens. Crop production is hampered by traditional farming methods, recurrent drought, erosion, and pests and diseases.

The invasive pest, fall armyworm (*Spodoptera frugiperda* J.E. Smith) (FAW), was confirmed as present in Botswana in 2017. Anecdotal studies in Botswana showed that in the 2017–2018 season, FAW posed a serious threat to food and nutrition security for vulnerable farming communities and households through reduction in harvest and increased production costs as a result of increased pesticides use.^10^ FAW is native to tropical America^11^, ^12^ where it is an important pest of maize and many other crops. Research studies in Africa have estimated that maize yield losses due to FAW in the range of 8.3 to 20.6 million tonnes per year, if management measures were not put in place.^13^ The pest has been reported to attack more than 350 plant species from 76 families.^14^ Although its larvae feed on a variety of plants, maize, peanuts, sorghum, millet and Bermudagrass are favoured hosts. Larvae usually consume a large amount of foliage and sometimes destroy the growing point of the plant.^15^ The most preferred hosts of FAW, maize, sorghum and millet, are however, the principal crops cultivated in Botswana and several other countries in Africa.

This study aimed to estimate impacts of FAW on yield and farmers' livelihoods in Botswana, and to obtain data that could be extrapolated to national level. Further, farmer knowledge of the pest, management practices and pesticide use for FAW management were assessed. This study will provide policy‐makers with data to make informed national action plans for the sustainable management and containment of the pest, and protect livelihoods.

## 2 MATERIALS AND METHODS

### 2.1 Study area

**Figure 1:**
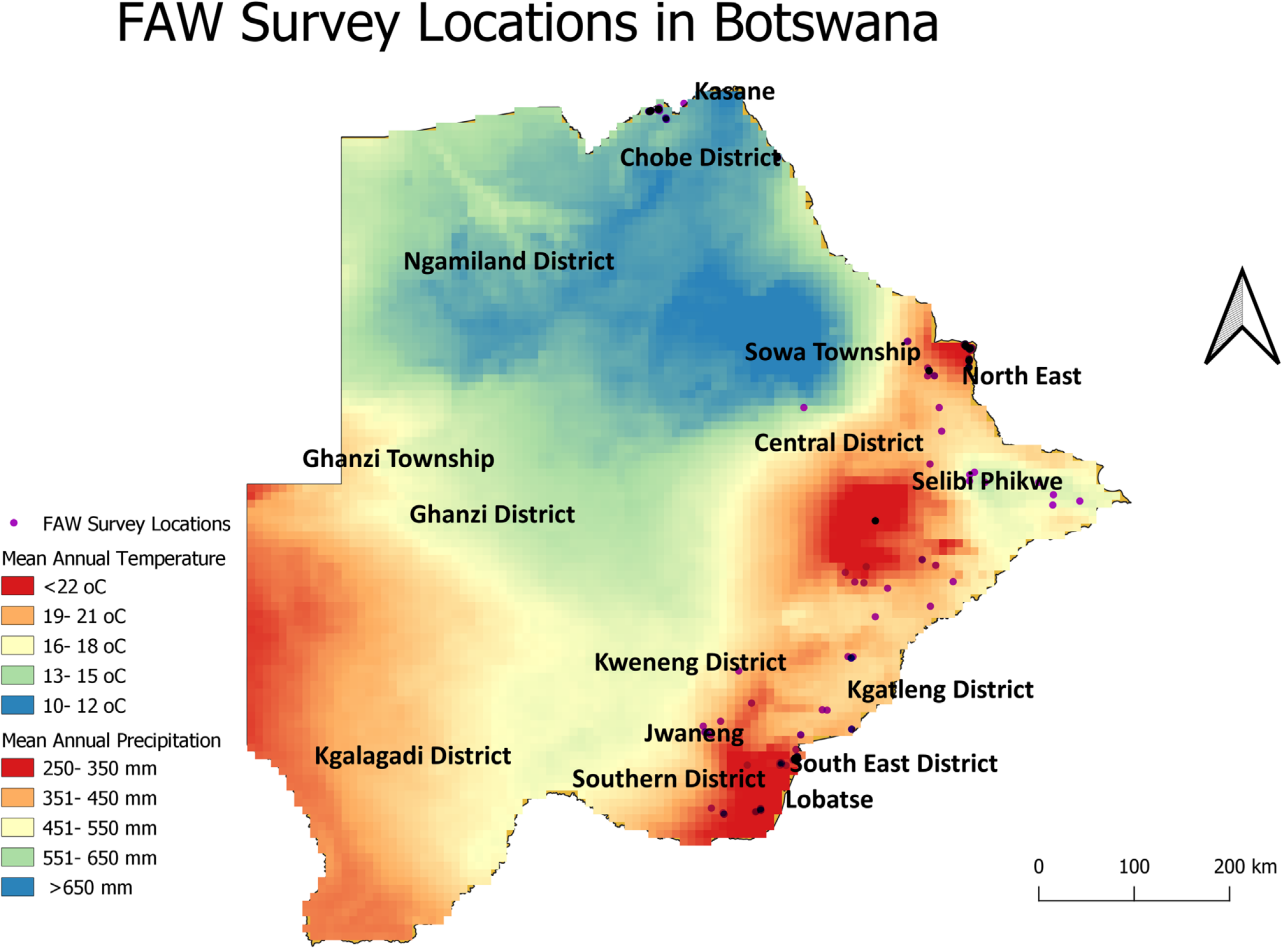
Map of FAW survey areas in Botswana.

The study targeted seven districts in Botswana (Table 1). Selection of districts was based on rapid assessment conducted by the Ministry of Agriculture that confirmed that the seven districts were affected by FAW. The target districts also represented different biophysical characteristics spanning mean annual temperature and precipitation of 10 to 24 °C and 250 to > 650 mm, respectively (Fig. 1).

**Table 1:** Sample size and distribution

No.	District	Sample frame	Target sample	Attained sample	Male	Female
1	Central	152	31	31	16	15
2	Chobe	425	86	81	31	50
3	Kgatleng	20	20	10	7	3
4	Kweneng	7	7	4	2	2
5	Northeast	771	155	61	25	36
6	Southern	23	23	16	13	3
7	Southeast	116	24	17	6	11
Total		1514	346	220	100	120

### 2.2 Household survey

A household survey was conducted in the target areas. The study population comprised of 1514 crop farming households in the seven districts. Using this population size, sample size was calculated using Krejcie and Morgan sample size determination method,^16^ at 95% degree of confidence and 5% margin of error. A total sample of 346 was obtained which was proportionately allocated to the seven study districts. Data were collected during February–March 2020. However, only 220 farm households were surveyed against the target of 346. Field data collection was affected by the Coronavirus disease (COVID‐19) situation in the country and indeed the rest of the world that led to restrictions in movements and social/physical distancing policies. Though the number of responses may reduce the power of this study, the team agreed to use the collected data, as it represented more than 10% of the target study population, and more than 100, the minimum sample required for a survey design.^16^

Data were collected using a questionnaire administered through face‐to‐face interviews by 13 trained enumerators. The questionnaire was converted into Open Data Kit (ODK) data, and data collection done using tablet computers. The survey targeted the household head or spouse, or any other member who was responsible for making farming decisions, and had adequate knowledge about the household. The questionnaire was pre‐tested before it was fully deployed. The surveys were conducted by CABI in partnership with Ministry of Agricultural Development and Food Security (MoA), Botswana. The survey tool captured information on household characteristics, farming activities, knowledge and perceptions of impacts of FAW on yield, and FAW control practices employed, shocks/stresses and needs/support. Farmer surveys are limited in providing extensive knowledge to understand farmer knowledge and practices for managing FAW, and there is a high possibility of overestimation of yield. However, in the absence of direct and systematic loss assessments and complimentary approaches, this method remains a practical approach to understand yield losses alongside management practices. Farmers' surveys have been successfully used in previous studies for estimating crop yield losses.^17^, ^18^

### 2.3 Analytical framework

The final datasets were downloaded from the aggregate server as CSV files and exported to STATA 15 software for analysis. Descriptive analysis was done by calculating frequencies, means, and standard errors. Chi‐squared (*χ* ^2^) and *t*‐tests were used to compare the significance of categorical variables and continuous data, respectively, by gender and FAW attack as reported by farmers. Regression model was used to estimate factors affecting crop yield. This can be expressed as:

(1) $$y_{i}=\alpha_{i}+\beta x_{i}+\varepsilon_{i}$$

where $y_{i}$ represents the yield of household i; $x_{i}$ is a vector of control variables, with the associated parameters β; and $\varepsilon_{i}$ is a random error term. Household characteristics, weather condition, FAW control practices, other production shocks, cropping pattern, farm size, presence of other pests, other variables that would predict yield such a severity of FAW, and crop condition, rainfall, and pest incidence were included as explanatory variables. Yield (in kg ha^−1^) was computed based on figures reported by farmers on production and area under cultivation for the 2018/2019 cropping season. Some farmers sold green maize, as a coping measure for FAW attack as well as the drought situation. In such a case, grain yield was estimated using Eqn (2):

(2) $$\text{Grain yield}=\left[\text{Fresh}\;\mathrm{ear}\;\text{weight}\times \text{shelling percentage}\right]\times \left(1-\%\text{moisture content}\right)$$

The estimated ear weight by farmers was 0.5 kg. Therefore, 80% shelling percentage and 20% moisture content were used based on the literature. This approach has been previously used in research,^19^, ^20^ though we acknowledge that shelling percentage can vary from one environment to another, as well as on the variety of maize. Only maize yield was included in the regression analysis as responses for other crops were very small, and therefore dropped.

However, the variables FAW control practice may be correlated with the error term (thus potentially an endogenous variables). This may arise from self‐selection where the farmers themselves decide whether or not to participate and implement management practices, probably due to differences in resource endowments, managerial ability, or motivation. To test for endogeneity of this variable, a Durbin–Wu–Hausman test was performed. Initially, we included a set of observable covariates – farm size and receiving FAW information – as instruments, and regressed the variable FAW control practice on its instrumental variables and the other parameters in the model. This was followed by a Durbin–Wu–Hausman test to test the hypothesis that the variable is exogenous.

## 3 RESULTS

### 3.1 Household characteristics

The majority of the respondents were farm managers (93%) but knowledgeable about farming decisions related to the target households. Only 7% of the respondents were either actual farm owners of spouses of the farm owner. The average age of farm owners as reported by the respondents was 59.5 years (Table 2). The average cultivated land per household was 9.5 acres [standard deviation (SD) = 28.1]. Male respondents reported a higher number of household members, including those working on farm either part time or full time. Maize, cowpeas, sorghum and millet were the principal crops cultivated, with 41% and 35% of female and male respondents, respectively, reporting that crop farming was their primary income source. In terms of proportion of food purchase to farming income, 36% of respondents reported to be spending about half (40–60%) of farming income on purchasing food. At least 21% of the respondents reported to using a major part (60–90%) of their farming income to purchase food while only 13% reporting to using a very minor part (< 10%) of their income to purchase food. Those who spent almost the entire farming income to purchase food were about 5%.

**Table 2:** Household profiles of farmers in Botswana

Variable	Overall	Female	Male
Age of household head (years)	59.5 (0.9)	57.9 (1.2)	61.5 (1.4)
Household size (#)	4.4 (2.8)	4.5 (3.1)	4.3 (2.4)
Household members full time on farm (#)	1.3 (1.3)	1.2 (1.3)	1.4 (1.3)
Household members part time on farm (#)	1.4 (2.0)	1.2 (1.6)	1.7 ** (2.4)
Household members in school (#)	2.1 (1.0)	2.1 (1.0)	2.2 (1.0)
Cultivated land (acres)	9.5 (28.1)	5.0 (9.3)	15.1 *** (40.3)
*Main crops (%)*			
Maize	87	86	89
Cowpeas	57	60	54
Sorghum	45	46	43
Millet	22	24	19
Crop farming primary source of income (%)	38	41	35
*Proportion of food purchase to income (%)*			
A very minor part (< 10%)	13	14	11
A minor part (10% to 40%)	24	24	25
About half (40% to 60%)	36	35	38
A major part (60% to 90%)	21	22	20
The entire or almost the entire income (> 90%)	5	5	6

Figures in parentheses are standard deviations.

*, ** and *** indicate that the difference is statistically significant at the 10%, 5% and 1% confidence levels, respectively.

### 3.2 Knowledge and perception of FAW infestation

The majority (76%) of the respondents had seen FAW in their farms (Table 3). Maize (84%) was the main crop attacked by FAW with an equal distribution between male and female respondents, while sweet reed was least affected. ‘Windowing’ effects (skeletonizing of leaves) as a result of FAW damage, was the most reported symptom during this survey. In terms of signs as exhibited by the presence of the pest (larvae or eggs), larvae were the most sign observed and reported (19%). FAW eggs were less observed by the respondents with only 2% of the farmers reporting the same. Other symptoms reported were damage near the tunnel, holes on maize cobs, caterpillar with ‘Y’ on the head and feeding on cobs by the larvae.

**Table 3:** General statistics for all fall armyworm (FAW) crops

Variable	Overall	Female	Male
Seen FAW on farm	76	73	80
*Crops affected by FAW*			
Maize	84	84	84
Sorghum	9	8	10
Cowpeas	5	8	2
Sweet reed	2	0	3
*FAW symptoms/development stages observed*	
Leaves with external feeding, creating ‘windowing’ effect	29	30	27
Larvae	19	19	19
Damage near the tunnel	14	14	13
Holes on maize cobs	12	10	14
Larvae within leaves with deep feeding	12	12	12
Caterpillar with Y on the head	8	9	8
Larvae feeding on cobs	5	5	5
Eggs	2	2	1
*Actions taken on FAW‐infested crops*	
Used as animal fodder	81	73	90
Used as compost	17	27	5
Burnt	2	0	5
*Season seen FAW for the first time*		
This cropping season (2018/2019)	43	43	44
A few cropping seasons ago (before 2016/2017)	31	29	34
The previous cropping season (2017/2018)	26	29	23
*Rainfall amount during season FAW was seen*	
Only light rainfall	62	57	68
Heavy rainfall	24	28	20
Dry spell	13	14	11

Figures are percentages within the male/female/overall categories. Chi square tests of independence did not show any statistically significant differences between respondent sex for all the variables presented on this table.

When asked what they did with the FAW affected crop, a majority (81%) of the respondents indicated that they used it as animal fodder. More female (27%) than male respondents (5%) reported to have used affected crops as compost with a very small proportion of respondents opting to burn the affected crops. During this survey it was also noted that 50% of respondents did not take any action on crops infested by FAW, opting to leave them in the field. A majority of the respondents reported to have seen FAW for the first time in the 2018/2019 cropping season. In terms of the impact of rainfall on presence or population of FAW, majority of respondents (62%) reported encountering FAW during light rainfall. A few reported to seeing FAW after heavy rain with a small number confirming to not seeing FAW during dry spell.

### 3.3 Perception of FAW infestation levels

For the most affected crops – maize and sorghum ‐ respondents were asked the crop stage that was most affected by FAW, when they observed it on their farms, and presence of other pests on the crop, and crop condition before FAW attack. This was aimed at understanding other underlying factors to FAW attack or incidence and therefore yield loss. A majority of the respondents reported that early vegetative stage was the most attacked for both maize (63%) and sorghum (71%). The same response was also captured for the late vegetative stage though with lower proportions reported − 16% and 12% for maize and sorghum, respectively. Generally, farmers reported less attack of the crop at harvest stage in maize (1%) but slightly more in sorghum (6%). There was no significant difference in the reported proportions between male and female respondents (Table 4).

**Table 4:** Fall armyworm (FAW) infestation on maize and sorghum

Variable (%)	Maize			Sorghum		
	Female	Male	Overall	Female	Male	Overall
*Stage affected most by FAW*						
Early vegetative	64	61	63	75	67	71
Late vegetative	20	12	16	13	11	12
Late maturity	5	15 **	10	0	11 ***	6
Early maturity	7	9	8	0	0	0
Emergence	4	1	3	13 ***	0	6
Harvest	0	1	1	0	11 ***	6
*Crop condition just before FAW attack*						
Very good plant condition	19	28	23	25 ***	0	12
Good plant condition	57 **	41	49	13	33 ***	24
Average plant condition	11	15	13	50	44	47
Poor plant condition	4	7	5	0	11 ***	6
Very poor plant condition	10	9	10	13	11	12
Crop suffered other diseases	0	11***	5	13 ***	0	6
Crop suffered other pests	21	31 *	26	25	22	24
						

Figures are percentages within the male/female/overall categories.

Note: *, ** and *** indicate that the difference is statistically significant at the 10%, 5% and 1% confidence levels, respectively.

Respondents were also asked if there were other pests observed on the crop during the growing season when they observed FAW. At least 26% and 24% reported presence of other pests on maize and sorghum, respectively. Other mentioned pests were stem borers, termites, bollworm, beetles, grasshoppers and wildlife. At least 5% and 6% of the farmers reported disease incidence on maize and sorghum, respectively. The main diseases reported were leaf blight, stalk rots and common smuts. In terms of crop condition, more than half of the respondents mentioned that maize crop was in very good or good condition before FAW attack, which for sorghum, a majority mentioned that the crop was in average condition.

Respondents were also asked to estimate the proportion of plants, and planted area affected by FAW when they observed it on their farms. At least 33% of the respondents reported that almost the entire area (> 90%) was affected by FAW (Table 5). For the sorghum farmers, 41% reported that a minor part (10–40%) of the land was affected by FAW. Of the farmers interviewed very few (4% for maize and 12% for sorghum) reported that a very minor part (< 10%) was affected by FAW. It is important to note for both maize and sorghum farmers, a bigger part of their lands (40–90%) were affected by FAW. In terms of proportion of plants affected, 31% and 41% of maize and sorghum farmers, respectively, reported a minor part (10–40%). FAW is able to fly to considerable distances aiding its spread within a farm in a short period of time. Indeed, this is reflected by the majority of farmers' (49% for maize and 24% for sorghum) responses that just before FAW attack their crops were in good condition.

**Table 5:** Farmer perception and estimate of fall armyworm (FAW) infestation levels on sorghum and maize

	Maize			Sorghum		
Variable (%)	Female	Male	Overall	Female	Male	Overall
*Area affected by FAW*						
The entire area (> 90%)	33	33	33	13	11	12
A minor part (10% to 40%)	26	25	26	38	44	41
About a half (40% to 60%)	25	23	24	13	44 ***	29
A major part (60% to 90%)	14	13	13	13 ***	0	6
A very minor part (< 10%)	2	5	4	25 ***	0	12
*Proportion of plants affected by FAW*						
A minor part (10% to 40%)	32	31	31	25	56	41
The entire area (> 90%)	26	27	26	0	11 ***	6
About a half (40% to 60%)	22	24	23	25	33	29
A major part (60% to 90%)	17	15	16	25 ***	0	12
A very minor part (< 10%)	2	4	3	25 ***	0	12

Figures are percentages within the male/female/overall categories.

Note: *, ** and *** indicate that the difference is statistically significant at the 10%, 5% and 1% confidence levels, respectively.

### 3.4 FAW management practices and perceived effectiveness

According to the household survey, just about half of the respondents (51%) reported that they implemented FAW control measures (Table 6). Significantly, male farmers (58%) however implemented FAW control measures compared to 45% female farmers. Eight different strategies were employed in the management of FAW, out of which chemical pesticides (27%) were the most common. Twice as many males (36%) than females (18%) used pesticides in the management of FAW in their farms. Use of ash/sand (13%) was the top non‐chemical control method against FAW reported by the farmers interviewed with more females (18%) using the method compared to males (10%).

**Table 6:** Fall armyworm (FAW) control methods used by farmers

Variable (%)	Overall	Female	Male	Effectiveness rating
Implemented FAW control measures (yes)	51	45	58*	
Pesticide	27	18	36***	2.1
Applying ash/sand in the funnel	13	15	10	2.0
Hand picking and crushing caterpillars/egg masses	7	8	6	1.9
Biological control measures	5	6	5	2.1
Removal/destruction of infected crop residue	3	0	6***	1.6
Frequent weeding	2	1	3	2.3

Figures are percentages within the male/female/overall categories.

Note: *, ** and *** indicate that the difference is statistically significant at the 10%, 5% and 1% confidence levels, respectively.

The data also showed that only 5% used biocontrol measures, 3% removed crop residues, 2% practiced frequent weeding, 1% replanted and 1% used neem‐based solutions. Across the sex categories, significance differences were observed in the use of pesticides and removal/destruction of infected crop residue (Table 6). Those who did not apply any control measures despite the observed infestation of FAW on their farm gave various reasons for inaction: pesticides being too expensive, inputs (pesticides) were too far and not easily accessible/available, weather conditions were not favourable, it was rainy and floods in some fields, it was too late to apply any control measures as the maize had matured, farmer did not know where to obtain the inputs, farmers did not manage to go to crop production officer for assistance, or did not understand how to apply the recommendations. Respondents were asked to rate effectiveness of the control measures used on a scale of 1 to 5, where 1 is ineffective and 5 very effective. The average effectiveness rating was two points implying below average effectiveness for all used methods.

### 3.5 Pesticide use

Various chemical compounds were used for the control of FAW in Botswana. Cypermethrin, chlorpyrifos and dimethoate were the most commonly used chemical compounds in the management of FAW (Table 7). According to the World Health Organization (WHO), these are all class II (moderately hazardous).

**Table 7:** Most commonly used pesticides for control of fall armyworm (FAW) and associated cost

Pesticide name (active ingredient)	WHO class	Percentage of farmers using pesticide	Rate (ml 20 L^−1^)	Frequency of spray in a season	Cost per hectare (pula)	Cost per hectare (US$)
Cypermethrin	II	33	20	1	103.0	8.7
Chlorpyrifos	II	8	12.5	2	65.4	5.5
Dimethoate	II	6	10	1	350.0	29.5
Deltamethrin	II	4	20	1.5	85.0	7.2
Carbaryl	II	4	5	1	173.3	14.6
Lambda‐cyhalothrin	II	2	60	1	120.0	10.1
Mercaptothion	III	2	20	1	120.0	10.1
Average			21	1.2	145.2	12.2
Median			20	1	120.0	10.1

1 pula = US$. 0.08421.

World Health Organization (WHO) classification: Ia = extremely hazardous; Ib = highly hazardous; II = moderately hazardous; III = slightly hazardous; U = unlikely to present acute hazard in normal use; n – not listed [list published in 2009 (WHO 2010)].^21^

The average cost per hectare was (145.25 pula, US$ 12.23; median = 120.0 pula, US$ 10.11) while the average cost per spray was US$0.16.59. In looking at the top two chemical compounds used, dimethoate and cypermethrin were the most expensive pesticides, and farmers spent on average 350 pula (US$0.29.47), 103 pula (US$. 8.7) per hectare, respectively. On average farmers sprayed 5.2 pumps (median = 5) per hectare in a single spray session and sprayed at least once (average = 1.4; median = 1) in the 2018/2019 cropping season. A majority (88%) of the respondents sourced the pesticides from agro‐dealers or from fellow farmers.

### 3.6 Shocks experienced

The survey sought to understand some of the shocks/stresses experienced by farmers during the 2018/2019 cropping season. A majority of the farmers (88.6%) experienced one or more shocks during the period under consideration with 11.4% reporting not experiencing any. The major shocks considered were: drought/insufficient water, excess rain/flooding, pest and diseases, hail, crop destruction by wildlife, and sickness (Table 8). These shocks were then ranked on a scale of 1 to 3; where 1 was the most important while 3 was the least important. Drought/insufficient water was ranked the major/important stress experienced by the famers interviewed (35%). Overall, pests and diseases (other than FAW) were ranked the second important stress by the farms. Other stresses mentioned by farmers were destruction by wildlife.

**Table 8:** Shocks/stress experienced by farmers during the 2019 cropping season

Shock/stress	Total	Male	Female
Drought/insufficient water	35	36	35
Excess rain/flooding	1	1	1
Pests/disease (other than fall armyworm)	15	17	13
Hail	0	0	0
Crop destroyed by livestock/theft	13	11	14
Sickness	0	0	0
Other, specify	11	11	11

Figures are percentages within the male/female/overall categories. Chi square tests of independence did not show any statistically significant differences between male and female respondents for all the variables presented on this table.

### 3.7 FAW effects on yield and livelihoods

The study assessed effects of FAW on crop yields and livelihoods in general. First, respondents were asked to estimate their maize and sorghum yields (crops mainly affected by FAW) in the 2018/2019 cropping season. Second, they were asked if the obtained food production was sufficient to last them to the next season, and third, if in ordinary times they faced similar food challenges due to abiotic or biotic stresses. The variables were compared between those who faced FAW and those that did not (Table 9). The average maize and sorghum yield during the 2018/2019 cropping season were 163 and 236 kg ha^−1^, respectively. The data shows differences in maize yield reported by farmers who did not experience FAW and those that did, with the latter reporting significantly lower yield values, while for sorghum yield, there was no observed difference. In terms of food self‐sufficiency, only 33% of respondents indicated that the harvested food would last them to the next season, as opposed to 80% in an ordinary year. Farmers who reported not to have been affected by FAW were more likely to be insufficient with food (88%) compared to 60% of the farmers who reported FAW attack.

**Table 9:** Yield and food self‐sufficiency indicators for households in the study locations

Variable	Full sample	Affected by fall armyworm	Not affected by fall armyworm
*Harvest in 2018/2019 cropping season (kg ha* ^*−1*^ *)*			
Maize	162.0 (26.5)	184.2 (31.1)	65.7* (37.9)
Sorghum	236.3 (38.5)	228.2 (39.5)	265.8 (108.0)
*Considering the recent harvest, will your food stocks last you up to the next season's harvest? (%)*			
Yes	33	40	12
No	67	60	88
Pearson *χ* ^2^(1)	11.65		
*P*	0.001		
*During an average year, would your own food stocks last you to the next season harvest? (%)*			
Yes	81	81	81
No	6	7	4
N/A, food produced mainly for sale	12	11	15
Pearson *χ* ^2^(2)	1.19		
*P*	0.552		

Figures in parentheses are standard error.

*, ** and *** indicate that the difference is statistically significant at the 10%, 5% and 1% confidence levels, respectively.

Regression analysis of factors affecting maize yield was done to understand effects of various explanatory variables (see Table 10). Indicators for abiotic and biotic factors such as crop condition, rainfall situation and presence of other pests and diseases were included as explanatory variables. Variance Inflation Factor (VIF) was used to test for multicollinearity of the independent variables. The VIF values were below 3 for all the variables implying that there was no multicollinearity amongst the selected variables. Durbin statistic and Wu–Hausman statistic test for endogeneity of FAW control practices showed insignificant *P*‐values, and thus the null hypothesis that the variable is exogenous was accepted.

**Table 10:** Factors affecting maize yield (kg ha^−1^)

Ln maize yield	Coefficient	Standard error
Respondent sex (1 = male)	−0.153	0.180
Weather condition: light rainfall	0.067	0.205
Weather condition: dry spell	−0.648**	0.308
Implemented FAW control practices (yes = 1)	0.473***	0.171
Experienced other shocks (yes = 1)	−0.503**	0.266
Maize cultivated area (ha)	0.002	0.003
Cropping system (monocrop =1)	−0.032	0.082
Presence of other pests (yes = 1)	0.488**	0.214
Presence of other diseases (yes = 1)	0.362	0.425
Proportional of area affected by FAW	−0.242***	0.071
Crop condition at the time of FAW attack	0.177**	0.089
Constant	1.680***	0.527
Observations	151	
*R* ^2^	0.3084	
*F*(12, 142)	5.19	
Prob > *F*	0.000	

Rainfall situation: base category is dry spell.

Proportion of area affected by fall armyworm (FAW) – ranked from 1 to 5, where 1 is a very minor part (< 10%) and 5 is the entire or almost the entire income (> 90%).

Crop condition = ranked from 1 to 5 where 1 is very poor and 5 very good plant condition.

*, ** and *** indicate that the difference is statistically significant at the 10%, 5% and 1% confidence levels, respectively.





Durbin (score) *χ*^2^(1) = 0.432776 (*P* = 0.5106)

Wu‐Hausman *F*(1,129) = 0.397163 (*P*= 0.5297)





Table 10 shows results of the regression analysis. The data shows that heavy rainfall and light rainfall were associated with a significant increase in maize yield compared to farmers who experienced dry spell during the season. Crop condition at the time of FAW attack was positively correlated with maize yield, that is a healthier crop was less likely to suffer FAW effects compared to one that was in poor condition. However, the proportion of crop affected by FAW was negatively associated with crop yield. Implementation of FAW control practices was positively correlated with maize yield.

### 3.8 Needs to prioritize in the next cropping season

In order to understand the needs/support farmers require going forward, the survey asked respondents to rank in order of importance (1 – most important; 3 – least important) what needs they would prioritize for the next season(s) (Table 11). Pesticides were the top ranked need (20%). Training on pest management was ranked second most need (18%). Other needs mentioned by farmers that ranked high included training on FAW management (7%); training on crop management (6%) and agricultural support services (7%). Among the support services mentioned include soil testing and sinking of boreholes to provide water for irrigation. It is worth noting that less than 1% of the farmers interviewed required marketing assistance. This could be probably because majority are subsistence farmers and only grow for subsistence use. Prioritizing farmers needs is an important approach in tailor‐making solutions. This information is important for agro‐dealers, extension service providers, researchers and policy‐makers. With knowledge of what farmers require, governments are able to formulate the right policy regulations, budget and allocate resources accordingly.

**Table 11:** Farmers' needs to prioritize for next season (%)

Needs	Overall	Male	Female
Awareness raising on fall armyworm	5	5	6
Training on pest management	13	13	14
Training on crop management	6	5	7
Training on fall armyworm management	7	4	10
Seeds	7	7	7
Pesticides	20	19	20
Fertilizer	3	4	3
Other agricultural inputs (please specify)	5	6	5
Agricultural tools (please specify)	8	10	6
Agricultural support services (please specify)	7	8	5
Marketing assistance	1	1	0
Other, specify	3	2	4

Figures are percentages within the male/female/overall categories.

*, ** and *** indicate that the difference is statistically significant at the 10%, 5% and 1% confidence levels, respectively.

## 4 DISCUSSION

### 4.1 Farmer knowledge of FAW and effects on livelihoods

The survey of farm households in Botswana showed that at least 76% of households observed FAW on their fields during the 2018/2019 cropping season, mainly on maize and sorghum. A few farmers indicated that they had observed the FAW on their field a season or two seasons before. The first official report of FAW presence in Botswana was made in 2017^22^ and as such, the responses of 2016/2017 cropping season confirm the record reports. Farmers reported more incidence of FAW during the dry spell compared to rainy season. Rainfall and other abiotic factors have an effect on pest population. The findings of this study concur with those of previous studies^23^ which reported that heavy rainfall can dislodge insects from the plants. In their study, they found a strong negative direct effect on the survival of *Plutella xylostella* (a lepidopteran herbivore) and increased development time of the pest by increased downpours. Farmers reported average maize and sorghum yields of 163 and 236 kg ha^−1^, respectively, during the 2018/2019 cropping season. Indeed this data relates to FAOSTAT data^24^ on maize yield in the country. The area harvested and total production (in tonnes) in 2018 was reported as: Maize (62 211 ha, 13 126 t); Millet (3273 ha, 1145 t) and Sorghum (27 584 ha, 17 835 t). Figures from the Annual Agricultural Survey Report in 2017 for Botswana's traditional sector are as follows: maize (51 000 ha, 14 000 Mt); millet (3000 ha, 1000 Mt) and sorghum (17 000 ha, 6000 Mt) for area harvested and production, respectively.^25^

However, this study finds that farmers who did not suffer FAW reported significantly lower yields for maize compared to those who reported FAW on their fields. This may imply other underlying abiotic and/or biotic factors that could have played an important role in determining yield. More so considering that a majority of farmers had experienced various production shocks, notable droughts. The regression results also indicate that farmers who experienced high rainfall or slight rainfall were more likely to have a better yield than those who experienced dry spells. This indicates that rainfall played a part in regulating the populations of FAW by washing them off and probably reducing the impact on the crop. Further, in contextualizing the portion of the crop affected, maize for instance is able to recover from FAW damage and as such the portion of crops affected referred to as ‘minor part’ could have been major but the crop was able to recover from the FAW damage possibly in response to management practices, e.g. pesticides spraying of FAW.

### 4.2 FAW control practices and implications for policy and practice

While various control measures have been studied, this study shows that farmers were not aware of available options for FAW management. The study also shows that the lack of action by some farmers was due to other environmental challenges faced by farmers at the time. Some respondents indicated that they did not find it necessary to control FAW because the maize crop was already performing poorly owing to lack of rain and the crop was at late maturity stage. It was also very likely that the reported yield was more affected by drought than FAW.

Majority of farmers based a large part of their FAW management practices on pesticides with a few using cultural and physical methods, based on indigenous knowledge, either in isolation or combination. This is consistent with previous studies^18^, ^26^, ^27^ which found out that smallholder farm households have adopted a variety of cultural, physical, chemical and local options to mitigate the effects of FAW, but the use of synthetic pesticides remains the most popular option. Some of these practices require validation to be scaled up. It was however noted that these practices were less effective in FAW management just as it has been reported in previous studies in Kenya and Ethiopia.^18^ Use of biopesticides was low as shown by the responses of this study. This is consistent with the study of Constantine *et al*.,^28^ in Kenya which reported on the low adoption/non‐use of biopesticides. In this study, perceptions of effectiveness, primarily speed of action and spectrum of activity, availability and affordability were the major reasons for low uptake. Biopesticides have been recommended as low‐risk alternatives to synthetic chemicals which negatively impact the environment, increase user cost, results in pest resurgence and pest resistance to insecticides.^29^

Several studies and projects have been conducted to demonstrate the efficacy of integrated pest management (IPM) in the management of FAW.^30^ In this regard, several options are proposed for consideration in FAW IPM programme. These include use of: biopesticides (botanicals and microbials), biological control (conservation of natural enemies, classical and inundative biocontrol), FAW pheromones (mating disruptors, mass trapping, etc.), host plant resistance, and judicious use of registered pesticides, with knowledge of the bio‐ecology of FAW. Four principles guide the implementation of an IPM programme and include: growing a healthy crop in a healthy farming system, conserving natural enemies, observing fields regularly and farmers becoming their own experts.^31^ It is proposed that the IPM programme be implemented on an area‐wide scale to minimize reintroduction of the pest into treated areas from untreated areas. Areawide pest management (AWPM) is defined as the ‘long‐term planned campaign against a pest insect population in a relatively large predefined area with the objective of reducing the insect population to a non‐economic status’.^32^ Currently, the control of many highly mobile and very destructive insect pests (e.g. FAW) is still carried out, for the most part, by individual producers who rely heavily on the use of insecticides. Although other control technologies are often incorporated into the producer's IPM system, these technologies, too, are usually applied by producers independently of other producers, and without due consideration of surrounding host and non‐host areas. Such uncoordinated farm‐by‐farm efforts often prove inefficient since they only suppress a proportion of the targeted pest population. Pests from nearby untreated areas remain unscathed and can re‐enter the treated areas, the damage continues, and people have little choice but to apply the control measures again and again to protect their livelihoods.

Thus, the key concept of area‐wide pest management is to address the whole pest population including all places of refuge or foci of infestation from which recruits could come to re‐establish damaging densities of the pest population in areas of concern. A key feature of this concept is that it is a long‐term campaign over a large geographical area, and not a one season activity. It provides a more cost‐effective and sustainable approach by proactively targeting entire pest populations. In this way, pest populations can be contained at low levels for longer periods and pest management methods can be integrated that are less reliant on pesticides and that better address ecological and environmental concerns. Although some of the methods deployed in AWPM may be costly and in some instances may not be affordable by individual farmers, when this strategy is deployed by a community, the *per capita* investment is lower compared to other conventional methods, and benefits accrue to all. To achieve this, training, mass awareness and capacity building activities will be required to package management messages to the right audience.

## 5 CONCLUSION

The results reported in this study are based on a combination of farmers' perception, infestation, their coping and management strategies, estimated crop/yield loss and needs to prioritize. A large proportion of the respondents had seen FAW in their farms in the current cropping season with about half of them practising some form of management. ‘Windowing’ was the most common symptom reported by farmers as a result of FAW larval feeding activity. Control of the pest was mainly based on pesticides. It is important to note that farmers in this study reported using other cultural and physical methods, based on indigenous knowledge, e.g. hand picking of egg masses and caterpillars, and application of ash/sand to the larvae in whorls as management of FAW. Even with low levels of efficacy, these methods provide alternative opportunities to expand the scope of an IPM programme. Validation and standardization by research are needed before wide‐scale promotion. FAW was reported to have an impact on yield and food self‐sufficiency. However, results from this study showed that those not affected by FAW were more likely to be food‐insufficient, indicating that more than one stress (other than FAW) was responsible for crop yield. Indeed, abiotic factors affect not just the crop production, including application of production and protection inputs, but also the development of pests. Rainfall levels resulted in a good crop condition which in turn had a positive correlation with yield at the time of FAW attack, that is, a healthier crop was less likely to suffer FAW effects compared to one that was in poor condition. Also, rainfall has been shown to regulate pest populations by washing them off plants. However, the proportion of crop affected by FAW was negatively associated with crop yield. Pesticides and training on pest management were the top prioritized needs for next seasons by the farmers.
